# Small Activating RNA Modulation of the G Protein‐Coupled Receptor for Cancer Treatment

**DOI:** 10.1002/advs.202200562

**Published:** 2022-06-16

**Authors:** Yunfang Xiong, Ran Ke, Qingyu Zhang, Wenjun Lan, Wanjun Yuan, Karol Nga Ieng Chan, Tom Roussel, Yifan Jiang, Jing Wu, Shuai Liu, Alice Sze Tsai Wong, Joong Sup Shim, Xuanjun Zhang, Ruiyu Xie, Nelson Dusetti, Juan Iovanna, Nagy Habib, Ling Peng, Leo Tsz On Lee

**Affiliations:** ^1^ Cancer Centre Faculty of Health Sciences University of Macau Taipa Macau 999078 China; ^2^ Department of Obstetrics and Gynaecology Affiliated Hospital of Guangdong Medical University Zhanjiang Guangdong 524001 China; ^3^ Aix Marseille Université CNRS Centre Interdisciplinaire de Nanoscience de Marseille (UMR 7325) Equipe Labellisée Ligue Contre le Cancer Marseille 13288 France; ^4^ Centre de Recherche en Cancérologie de Marseille (CRCM) INSERM U1068 CNRS Aix‐Marseille Université and Institut Paoli‐Calmettes Marseille 13288 France; ^5^ School of Biological Sciences The University of Hong Kong Pokfulam Road Hong Kong China; ^6^ MOE Frontiers Science Center for Precision Oncology University of Macau Taipa Macau 999078 China; ^7^ Department of Surgery and Cancer Imperial College London London W12 0NN UK; ^8^ MiNA Therapeutics, Translation & Innovation Hub 80 Wood Lane London W12 0BZ UK; ^9^ Centre of Reproduction, Development, and Aging Faculty of Health Sciences University of Macau Taipa Macau 999078 China

**Keywords:** cancer therapies, dendrimer vectors, G protein‐coupled receptors, Mas receptors (MAS1s), small activating RNA

## Abstract

G protein‐coupled receptors (GPCRs) are the most common and important drug targets. However, >70% of GPCRs are undruggable or difficult to target using conventional chemical agonists/antagonists. Small nucleic acid molecules, which can sequence‐specifically modulate any gene, offer a unique opportunity to effectively expand drug targets, especially those that are undruggable or difficult to address, such as GPCRs. Here, the authors report  for the first time that small activating RNAs (saRNAs) effectively modulate a GPCR for cancer treatment. Specifically, saRNAs promoting the expression of Mas receptor (MAS1), a GPCR that counteracts the classical angiotensin II pathway in cancer cell proliferation and migration, are identified. These saRNAs, delivered by an amphiphilic dendrimer vector, enhance MAS1 expression, counteracting the angiotensin II/angiotensin II Receptor Type 1 axis, and leading to significant suppression of tumorigenesis and the inhibition of tumor progression of multiple cancers in tumor‐xenografted mouse models and patient‐derived tumor models. This study provides not only a new strategy for cancer therapy by targeting the renin‐angiotensin system, but also a new avenue to modulate GPCR signaling by RNA activation.

## Graphical abstract

Small activating RNAs (saRNAs) targeting MAS1, delivered by an amphiphilic dendrimer (AD) vector, enhance MAS1 expression and counteract the angiotensin II pathway in cancer cells, leading to significantly suppressed tumorigenesis and inhibited tumor progression. This saRNA approach opens a new avenue of using saRNA to target undruggable G protein‐coupled receptors for drug development and disease treatment.

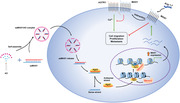

## 1 Introduction

G protein‐coupled receptors (GPCRs) are important pharmacological targets in drug discovery and development. Approximately 34% of drugs approved by the U.S. Food and Drug Administration (FDA) target GPCRs.^[^ ^1^ ^]^ However, these FDA‐approved drugs only target around 27% of the non‐olfactory GPCRs, meaning that a large number of GPCRs are currently “non‐druggable.” Furthermore, the specificity of conventional chemical agonists/antagonists of GPCRs is a major concern in drug development due to the various subtypes and splice variants as well as the high similarity of GPCRs within the same subfamily and target selectivity. Therefore, a new strategy is urgently needed to develop effective and specific drug candidates to expand the druggable “GPCRome.”

Small nucleic acid molecules, such as small interfering RNAs (siRNAs) and small activating RNAs (saRNAs), can target any gene and modulate gene expression in a potent and sequence‐specific way via Watson–Crick base pairing.^[^ ^2^ ^]^ This approach offers unique advantages to address novel and difficult drug targets, including the undruggable GPCRs. The first siRNA drug patisran (Onpattro), approved by FDA in 2018 for hereditary transthyretin‐mediated amyloidosis,^[^ ^3^ ^]^ has opened a new era of small RNA therapeutics. In contrast to siRNA, which inhibits gene expression,^[^ ^4^ ^]^ saRNA is a genetic approach to enhance gene expression.^[^ ^5^ ^]^ Similar to siRNA in structure, saRNA is also a double‐stranded RNA molecule with a short sequence of 19–21 base‐pairs. Specifically, saRNA can recruit the RNA‐induced transcriptional activation complex, in which the sense strand (also called the passenger strand) is discarded, and the remaining antisense strand (or guide strand) is paired with the promotor region of the target gene, stimulating transcription and leading to transcriptional activation of the proximal gene.^[^ ^6^ ^]^ Currently, the first saRNA drug (MTL‐CEBPA) has been evaluated in advanced liver cancer and demonstrated clinical proofs of targeting, and upregulating, the master myeloid transcription factor CCAAT enhancer‐binding protein *α* (CEBPA).^[^ ^7^ ^]^

Although the use of siRNA in inhibiting GPCR signaling is widely proposed, there has not been a report on RNA activation to modulate GPCR until now. In the present study, we have explored the use of GPCR‐targeting saRNAs for their potential in cancer therapy. The target GPCR is the Mas receptor (MAS1), which modulates the well‐known renin‐angiotensin system (RAS). The RAS plays a fundamental role in multiple physiological systems and is involved in cancer progression and metastasis.^[^ ^8^ ^]^ Dysregulation of RAS is associated with pathological conditions, and impacts on malignancy of tumors via tissue remodeling, inflammation, angiogenesis, and apoptosis.^[^ ^8^ ^]^ Hence, targeting RAS has been proposed as a novel approach to control the tumor microenvironment as well as tumor growth and dissemination.^[^ ^9^ ^]^ saRNAs that specifically and precisely regulate MAS1 expression could therefore offer a promising approach for treating cancer.

Practical implementation of RNA therapeutics requires safe and effective RNA delivery,^[^ ^2^, ^10^ ^]^ as RNA molecules are unstable and easily degraded via chemical and enzymatic hydrolysis. In addition, RNA molecules are hydrophilic and polyanionic and hence cannot readily penetrate the cell membrane and reach the cell cytoplasm. To achieve efficient delivery of saRNAs targeting MAS1 in this study, an “amphiphilic dendrimer” (referred to “AD” hereafter) has been employed as the delivery system. AD is an effective siRNA delivery vector previously developed by us,^[^ ^11^ ^]^ and it leverages the delivery advantages of the widely used lipid and polymer vectors. AD can protect small RNA molecules from degradation, shield the negative charge of RNA, and promote transport across the cell membrane for efficient delivery.

In this study, we demonstrate that MAS1 saRNAs (saMAS1) delivered by AD consistently enhanced the expression of *MAS1* in the RAS system in multiple cancer cell lines and models. The increase in *MAS1* expression resulted in effective inhibition of cancer cell migration and had a potent anticancer effect, leading to significant suppression of tumorigenesis in xenograft models and reduced growth of patient tumor‐derived organoids. Our studies demonstrate that saRNA‐mediated increase of *MAS1* expression is an effective approach to inhibit cancer proliferation in patient‐derived tumor models and should have broad relevance in cancer treatment. To our knowledge, this is the first report of using saRNA to target a GPCR. Our proof‐of‐concept study using MAS1 saRNA delivered by the dendrimer vector to regulate RAS not only validates the regulation of GPCR signaling via the saRNA approach but also provides a perspective for novel saRNA‐based therapeutics for undruggable targets in general.

## 2 Results

### 2.1 Specific saRNAs Effectively Increase MAS1 Expression

**Figure 1:**
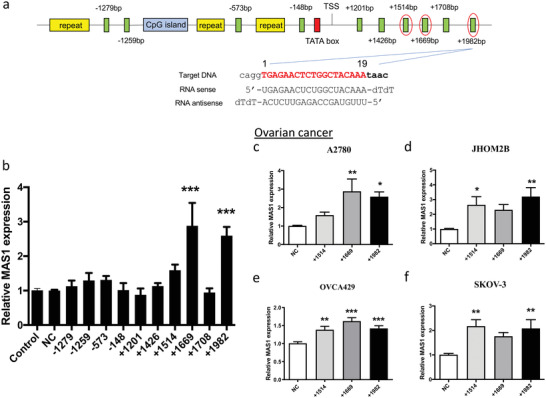
Design and validation of saRNAs to enhance MAS1 expression in ovarian cancer cells. a) Schematic diagram of the location of MAS1 saRNAs in the promoter region of the *MAS1* gene. Green boxes represent the tested saRNAs. The CpG island (blue box), repeat elements (yellow boxes), and TATA box (red box) were avoided in the saRNA design. The sequence of one MAS1 saRNA (+1982 bp) is shown in detail. The saRNAs selected for further analysis are highlighted by red circles. b) Screening of saRNAs for upregulated *MAS1* mRNA expression in A2780 cells. Ten different saRNAs (50 nm) were transfected into A2780 cells with Lipofectamine 3000. Relative expression was determined by real‐time qPCR. c–f) Upregulation of *MAS1* mRNA expression by saMAS1+1514, +1669, and +1982 in ovarian cancer c) A2780, d) JHOM2B, e) OVCA429, and f) SKOV‐3 cells. Cells were treated with saMAS1 (50 nm) and Lipofectamine 3000. All the data in this figure are presented as means ± SEM values from ≥3 experiments. *p* values are calculated by using one‐way ANOVA with Dunnett correction. Significant differences are indicated as *p < 0.05; **p < 0.01; ***p < 0.001 versus control.

To activate *MAS1* expression, a series of saRNA oligonucleotides were designed and named by their relative positions with respect to the transcription start site (**Figure** **1**a). These saRNA molecules were delivered into human ovarian cancer A2780 cells using the commercial vector Lipofectamine to evaluate their potential effects on *MAS1* expression. Our initial screening identified three saRNAs that upregulated MAS1 transcript levels: both saMAS1+1669 and saMAS1+1982 increased *MAS1* transcription significantly (by approximately threefold), whereas a slight increase was found with saMAS1+1514 (Figure 1b). The effects of these three saRNAs were verified in the following four different ovarian cancer cell lines: A2780, JHOM2B, SKOV‐3, and OVCA429 (Figure 1c–f). Notably, gene activation was also associated inversely with the level of endogenous gene expression in the cell lines (Figure S1, Supporting Information). For A2780 cells, which had the lowest endogenous expression of *MAS1*, all three saRNAs enhanced gene expression significantly and achieved a greater fold change than those seen in the tested cell lines. Among the three saRNAs, saMAS1+1982 upregulated *MAS1* expression consistently in all cell lines tested.

### 2.2 Dendrimer Vector AD Effectively Delivered saRNAs and Promoted MAS1 Expression

As the commercial vector Lipofectamine is toxic and not biocompatible for biological evaluation in animal models, we used the dendrimer vector AD to deliver saMAS1. AD was proved previously to be effective and biocompatible for siRNA delivery in vitro in various cells including cancer cells, primary immune cells, and stem cells as well as in vivo in different animal models.^[^ ^11^, ^12^ ^]^ Similarly as with siRNA, the dendrimer vector AD was able to form robust complexes with the saRNA, protect it against enzymatic degradation, and promote its cellular uptake.

**Figure 2:**
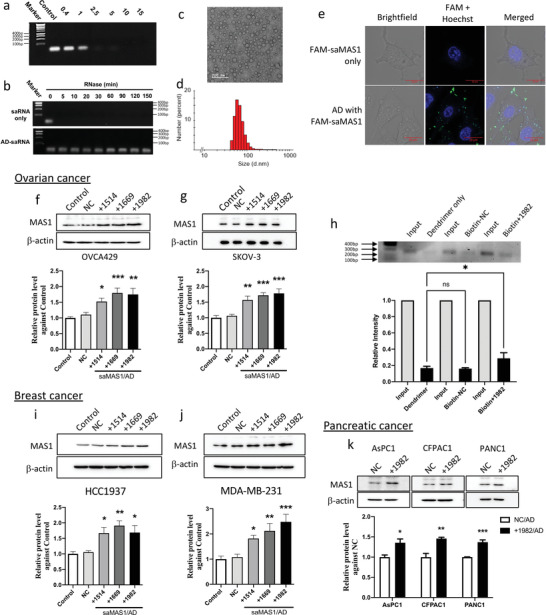
Dendrimer vector AD forms nanoparticles with saRNA and delivers saMAS1 in ovarian, breast, and pancreatic cancer cells for enhancing *MAS1* gene expression. a) Agarose gel‐shift analysis suggests the interaction of saMAS1+1982 with AD and complete encapsulation of saRNA at the N/P ratios of ≥2.5. b) The saRNA/AD complexes protect saMAS1+1982 from RNase A digestion. c) TEM imaging and d) DLS analysis of saMAS1+1982/AD complexes at an N/P ratio of 10. e) Confocal fluorescence images of FAM‐labeled saMAS1+1982 demonstrate the delivery of saRNA by AD into SKOV‐3 cells. The cells were treated with FAM‐labeled RNA complexed with AD, then stained with Hoechst 33342 before imaging. Blue: Hoechst 33342; green: FAM‐labeled RNA. f,g) saMAS1/AD increases the MAS1 protein levels in the ovarian cancer cell lines f) OVCA429 and g) SKOV‐3 as evaluated by western blotting. Upper: representative western blot; lower: quantification of protein levels from three experiments. h) The ChIbRP assay confirmed the binding of biotinylated MAS1 saRNA+1982 to the *MAS1* gene. Upper: agarose gel image of the PCR product from the input control and the *MAS1* chromatin following pull‐down by biotinylated saRNA; lower: the relative levels of PCR products compared to the input control. i,j) Three saMAS1/AD complexes (+1514, +1669, and +1982) enhanced the MAS1 protein levels in breast cancer i) HCC1937 and j) MDA‐MB‐231 cells as determined by western blotting. k) saMAS1+1982/AD enhanced MAS1 protein levels in pancreatic cancer cell lines (AsPC1, CFPAC1, and PANC‐1). All data in this figure are presented as mean ± SEM values from ≥3 experiments. *p* values are calculated by using one‐way ANOVA with Dunnett correction. An unpaired t‐test was used for two‐group comparisons in (k). Significant differences are indicated (*p < 0.05; **p < 0.01; ***p < 0.001 vs control; ns, not significant).

As shown in **Figure** **2**a, AD retarded saRNA migration in an agarose gel, indicating formation of stable saRNA/AD complexes at N/P ratios >2.5. The N/P ratio refers to the ratio of the number of amine terminals (N) in AD relative to the number of phosphate moieties (P) in the saRNA. Importantly, the saRNA/dendrimer complex protected saRNA from degradation by RNase. The complexed saRNA remained intact even after 120 min, whereas naked saRNA was rapidly decomposed within 5 min by RNase (Figure 2b). AD also protected the saRNA from degradation in the serum (Figure S2a, Supporting Information), suggesting that the protection effect is consistent in serum. Importantly, the saRNA/AD complexes were small and spherical, that is, ≈40 nm in size, as revealed by transmission electron microscope (TEM) imaging (Figure 2c) and dynamic light scattering (DLS) analysis (Figure 2d). All these features of the saRNA/AD complexes are favorable for their cell uptake.

To verify the cellular uptake of saRNA, the saMAS1+1982 RNA was fluorescently labeled by FAM or Cy3. Fluorescence imaging confirmed that FAM or Cy3 labeled saMAS1+1982 complexed with AD could successfully enter the SKOV‐3 and PANC‐1 cancer cells after a 4 h incubation, whereas no or little uptake was observed with the labeled saRNAs in the absence of AD (Figure 2e and Figure S2b,c, Supporting Information). The uptake of the saMAS1/AD was further confirmed by flow cytometry analysis (Figure S2d–g, Supporting Information). Collectively, these results highlight that AD successfully protects the saRNA from degradation and enables effective uptake into mammalian cells.

Having confirmed the promising features of the AD vector for complexing with saRNA and promoting its cellular uptake, we next assessed the functional delivery of saMAS1. In all tested ovarian cancer cell lines (A2780, JHOM2B, SKOV‐3, and OVCA429), *MAS1* RNA expression was enhanced when the three identified saRNAs (saMAS1+1514, saMAS1+1669, and saMAS1+1982) were, respectively, delivered by AD (Figure S3a–d, Supporting Information). This was in line with our findings obtained using Lipofectamine as the transfection reagent. The corresponding increase in the expression of MAS1 protein upon treatment with saRNA/AD was also examined and confirmed using western blotting (Figure 2f,g and Figure S4a,b, Supporting Information). Consistent with changes in transcript levels, saMAS1+1669 and saMAS1+1982 had the strongest MAS1 protein levels.

To confirm the interaction of saRNA‐activating complex with the target *MAS1* promoter, we performed a chromatin isolation by biotinylated RNA pull‐down (ChIbRP) assay. We used biotinylated saRNA to precipitate the chromatin that was associated with saMAS1+1982 binding after AD‐mediated delivery into A2780 cells (Figure 2h). Polymerase chain reaction (PCR) amplification of the *MAS* gene targeting the position flanking the location of saMAS1+1982 (from +1925 to +2172) resulted in a prominent specific signal in saRNA/AD‐treated cells. Compared to controls composed of the vector AD alone and biotinylated non‐target saRNA (Biotin‐NC), the biotinylated saRNA significantly increased the intensity of the pulled‐down *MAS1* fragment. This assay confirmed the interaction of saMAS1+1982 with the *MAS1* gene. To further demonstrate the specificity of saRNA, the expression levels of nearby genes (including *PNLDC1*, *TCP1*, *MRPL18*, *ACAT2*, and *IGF2R*) were measured, and no significant changes in their expression were observed (Figure S5, Supporting Information). Collectively, these data indicate that saMAS1+1982 is highly specific to the target gene.

The efficiency of the saMAS1/AD system was further assessed in breast cancer cells (HCC1937 and MDA‐MB‐231) and pancreatic cancer cells (AsPC1, CFPAC1, and PANC1). Compared to the untreated and non‐target (NC) controls, all three MAS1 saRNAs delivered by AD significantly enhanced the MAS1 protein (Figure 2i,j) and transcript (Figure S6a,b, Supporting Information) levels in both breast cancer cell lines. Further, a significant increase in MAS1 protein was detected in all tested pancreatic cancer cell lines (AsPC1, CFPAC1, and PANC1) after saMAS1+1982/AD treatment (Figure 2k). These data clearly demonstrate that the application of saRNAs targeting the GPCR MAS1 can increase its RNA and protein expression levels across multiple cancers.

### 2.3 MAS1 Suppressed the Angiotensin II Receptor Type 1 Signaling Pathway via Formation of a Receptor Heterocomplex

MAS1 has been shown to counteract the angiotensin II receptor type 1 (AGTR1)‐mediated signaling pathway in RAS on multiple physiological systems,^[^ ^13^ ^]^ including malignant tumors.^[^ ^8^ ^]^ Recent evidence suggests that the inhibitory activity induced by MAS1 against AGTR1 may be independent of the proposed endogenous ligand Ang 1–7.^[^ ^14^ ^]^ Thus, we investigated how the increase in MAS1 levels might counteract AGTR1 activation in a ligand‐independent manner.

**Figure 3:**
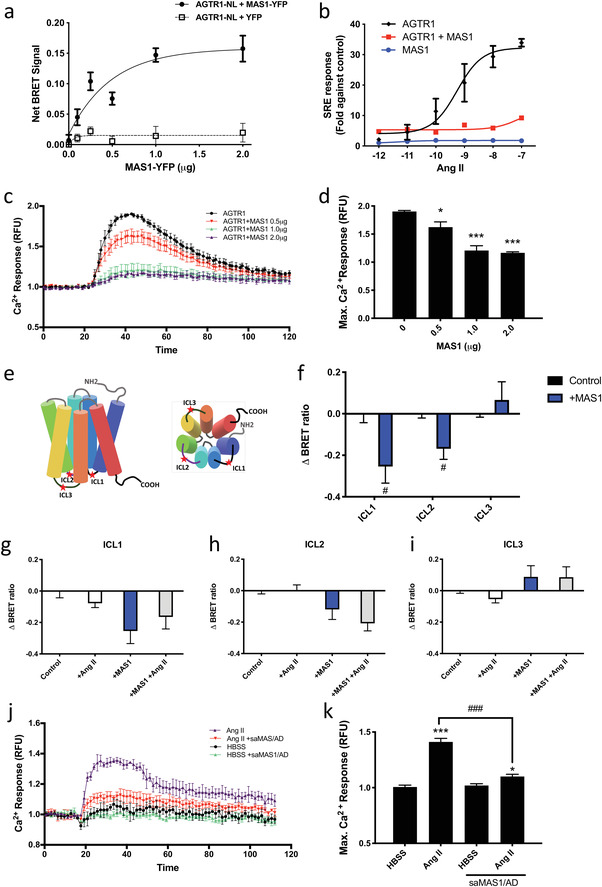
MAS1 inhibits the activation of AGTR1 by formatting a heterocomplex. a) Saturation BRET assays of MAS1 and AGTR1 interaction. A fixed amount of luciferase‐tagged AGTR1 (AGTR1‐NL, 0.25 µg) was co‐expressed with increasing amounts of YFP‐tagged MAS1 (MAS1‐YFP, 0–2.0 µg) in HEK293 cells. The total amount of transfected DNA was balanced with the pcDNA3.1 vector. The association curve of BRET signal reflects a specific protein–protein interaction. b) In HEK293 cells, co‐expression of MAS1 inhibited AGTR1‐mediated Ang II signaling in the SRE pathway. For the control experiments, AGTR1 or MAS1 were co‐transfected with pcDNA3.1. c) In HEK293 cells, the calcium response induced by AGTR1 was impaired by co‐expression of MAS1. Ang II (100 nm) was added at 20 s; signals between 0 and 20 s were defined as the baseline. d) Comparison of the maximum calcium response when different amounts of MAS1 were co‐transfected. e) Schematic diagram showing the location of tetracysteine tag sequence (CCPGCC) tags on AGTR1. f–i) The intramolecular net BRET signal was measured in HEK293 cells transfected with the indicated constructs. Cells were stimulated with or without Ang II (100 nm) as indicated. j,k) In OVCA429 cells, saMAS1/AD treatment significantly reduced the calcium response that induced by Ang II. All data in this figure are presented as mean ± SEM values from ≥3 experiments. *p* values were calculated by the GraphPad Prism software (GraphPad Software). *p < 0.05; ***p < 0.001 versus control (one‐way ANOVA); #*p* < 0.05 versus control (unpaired t‐test); ###*p* < 0.001 versus HBSS control (one‐way ANOVA).

GPCR dimerization can modulate receptor function, including cellular signaling. Therefore, we hypothesized that the increase in MAS1 levels suppresses AGTR1 activation directly via the formation of MAS1/AGTR1 heterocomplexes. We studied the interaction between MAS1 and AGTR1 in HEK293 cells using saturation bioluminescence resonance energy transfer (BRET) (**Figure** **3**a). Our results show a progressive increase in BRET signals in a hyperbolic manner, indicating a specific interaction between AGTR1 and MAS1.

We therefore first studied the effects of MAS1 on the AGTR1 signaling pathway using luciferase‐based serum responsive element (SRE) and calcium assays in HEK293 cells, where we observed that MAS1 co‐expression dramatically suppressed the SRE response of AGTR1 (Figure 3b). A similar result was revealed in the calcium assay: MAS1 co‐expression significantly inhibited the AGTR1‐mediated intracellular calcium pathway in a dose‐dependent manner (Figure 3c,d).

To determine whether the interaction with MAS1 modulated the conformation of AGTR1, we measured the distance between the intracellular loops (ICLs) of AGTR1 and the C‐terminal luciferase tag. To do this, we employed an intramolecular BRET assay, as was used previously to detect conformational changes in AGTR1 upon ligand binding.^[^ ^14^ ^]^ A tetracysteine tag (CCPGCC) was incorporated into each loop (ICL1, ICL2, or ICL3) of AGTR1 for fluorescein arsenical hairpin (FlAsH) labeling (Figure 3e). Co‐expression of MAS1 with the three tagged ICL variants of AGTR1 significantly altered the BRET signals in the absence of a ligand (Figure 3f). This finding suggests that the interaction of MAS1 can induce a conformational change in AGTR1 and, hence, alter the distance between the ICLs and the C‐terminal luciferase. Furthermore, treatment with Ang II did not reverse this conformational change in any of the ICL variants (Figure 3g–i), indicating that ligand binding cannot convert the AGTR1 into an active conformation after the formation of the heterocomplex.

To verify the effect of saMAS1/AD on cancer cells, we tested the calcium response in OVCA429 cells after saMAS1/AD (+1982) treatment (Figure 3j,k). The results indicate that saMAS1/AD significantly reduced the Ang II‐mediated calcium response and confirm that saMAS1/AD can modulate GPCR signaling in cancer cells. Taken together, our data offer molecular evidence that the increase in MAS1 expression is able to suppress the AGTR1‐mediated SRE activity and the calcium response via formation of receptor heterocomplexes in cancer cells.

### 2.4 saMAS1 Suppressed Cell Migration and Enhanced Endoplasmic Reticulum Stress in Cancer Spheroids

**Figure 4:**
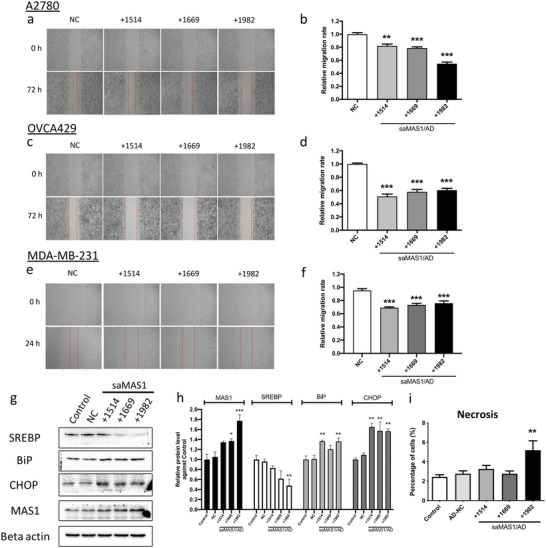
saMAS1/AD suppresses the migration of cancer cells and increases ER stress in spheroids. a–f) Wound healing assay for three different saMAS1/AD formulations in ovarian cancer A2780 cells (a,b), OVCA429 cells (c,d), and breast cancer MDA‐MB‐231 cells (e,f). The green lines represent the starting lines; the red lines represent the cells that had migrated by the end of the study period. The cells were scratched after 24 h of treatment with all three saMAS1s separately delivered by AD. a,c,e) Representative images of the assay. b,d,f) Relative migration rates in different samples. g) Effects of saMAS1/AD formulations on ER stress. Protein levels of SREBP (a transcription factor that regulates lipogenic enzymes), BiP and CHOP (ER stress markers), and MAS1 (the saRNA‐targeted receptor) were quantified by western blotting. h) The relative band intensities of the western blots are shown. i) Spheroids were generated from OVCA429 cells, then treated with the indicated saMAS1/AD formulations. Cell necrosis inside the spheroids was detected by flow cytometry. In all experiments, the final saMAS1 concentration was 50 nm, with AD at an N/P ratio of 10. All data are presented as mean ± SEM values from ≥3 experiments. *p* values are calculated by using one‐way ANOVA with Dunnett correction. Significant differences are indicated as *p < 0.05; **p < 0.01; ***p < 0.001 versus controls.

MAS1 has been reported to counteract AGTR1‐mediated cancer development and metastasis.^[^ ^15^ ^]^ We therefore examined the effects of saMAS1/AD on cell motility using a wound‐healing assay in two ovarian cancer cell lines (A2780 and OVCA429) treated with all three identified saRNAs, respectively, delivered by AD. At 72 h after scratching, all three saMAS1/AD complexes significantly inhibited the migration of A2780 and OVCA429 cells (**Figure** **4**a–d). Consistent with the levels of gene activation and protein expression, saMAS1+1982/AD showed the strongest inhibition in A2780 cells, while all saRNAs showed similar effects on OVCA429 cells. Similarly, all three saMAS1/AD complexes repressed the migration of MDA‐MB‐231 breast cancer cells (Figure 4e,f). Together, these data suggest that upregulation of MAS1 expression by saMAS1 effectively inhibits cancer cell migration.

AGTR1 activation leads to enhanced lipid desaturation and suppression of endoplasmic reticulum (ER) stress to prevent cell death in cancer spheroids.^[^ ^15^ ^]^ Therefore, we further evaluated the potential effects of saMAS1/AD in counteracting AGTR1 to promote ER stress in spheroids derived from OVCA429 cells. saMAS1/AD significantly decreased the expression of a key lipid metabolism protein, sterol regulatory‐element binding protein (SREBP), while two markers of ER stress, binding immunoglobulin protein (BiP) and C/EBP homologous protein (CHOP), were significantly increased in ovarian cancer spheroids following treatment with saMAS1/AD complexes (Figure 4g,h). Moreover, the number of necrotic cells increased in ovarian cancer spheroids (Figure 4i and Figure S7, Supporting Information). These results suggest that upregulation of MAS1 expression by saMAS1 effectively increases ER stress and induces cell death in addition to inhibiting cell migration.

### 2.5 saMAS1/AD Suppressed Tumorigenesis and Tumor Growth in Xenograft Models and Patient‐Derived Organoids

**Figure 5:**
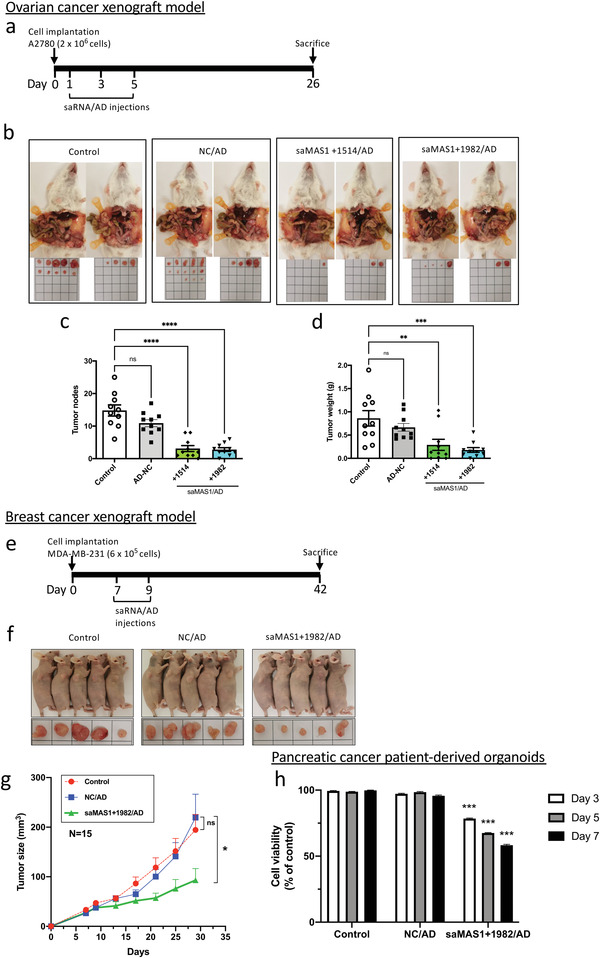
Effects of saMAS1/AD on ovarian and breast cancer xenograft models and patient pancreatic tumor‐derived organoids. a) Timeline showing establishment of a xenograft mouse model using A2780 ovarian cancer cells and the administration of saMAS1/AD to animals. b) Effects of different treatments on the ovarian cancer xenograft model. Administration of saMAS1+1514/AD and saMAS1+1982/AD (saMAS1 at 1.0 mg kg^−1^ and AD at N/P = 5) greatly reduced the development of A2780‐derived tumors within the peritoneal cavity of mice. Tumors excised from the peritoneal cavity are shown for each group (*n* = 10). c,d) The number of tumor nodes and the weights of the excised tumors from each group. Significant differences are indicated as **p < 0.01; ***p < 0.001; ****p < 0.0001; ns, not significant versus control. e) Timeline showing establishment of a xenograft mouse model using MDA‐MB‐231 breast cancer cells and administration of saMAS1+1982/AD to animals. f) Effects of different treatments on the breast cancer xenograft model. Administration of saMAS1/AD (saMAS1 at 1.0 mg kg^−1^ and AD at N/P = 5) greatly reduced the development of MDA‐MB‐231‐derived tumors in xenograft mice. Representative images of the xenograft mice and the excised tumors. g) Tumor size changes in the breast cancer xenograft model (*n* = 15), *p < 0.05; ns, not significant versus NC/AD. h) Treatment with saMAS1+1982/AD significantly reduces the viability of cells in organoids derived from pancreatic cancer patients. No significant changes were found in the groups treated with NC control RNA. Significant differences are indicated as ***p < 0.001 versus control. All data in this figure are presented as mean ± SEM values. *p* values are calculated by using one‐way ANOVA with Dunnett correction.

The effect of saMAS1 on tumorigenesis was first assessed using NOD/SCID mice xenografted with ovarian cancer A2780 cancer cells. Specifically, saMAS1+1514 and saMAS1+1982 were administered at 1.0 mg kg^−1^ after complexing it with the dendrimer vector AD at an N/P ratio of 5. In total, mice received three injections of saMAS1/AD complexes on days 1, 3, and 5 (**Figure** **5**a). Both saMAS1+1514/AD and saMAS1+1982/AD significantly and dramatically suppressed tumorigenesis and tumor growth in the xenograft model (Figure 5b). Following treatment with saMAS1+1982/AD, the number and weight of tumors decreased by 82% and 85%, respectively (Figure 5c,d). Data from the xenograft model are consistent with the in vitro data shown in Figure 4 and provide further evidence that saMAS1/AD can suppress the tumorigenesis and tumor progression of ovarian cancer.

The anticancer activity of saMAS1/AD was further verified using a breast cancer xenograft model with MDA‐MB‐231 cells (Figure 5e). Treatment with saMAS1+1982/AD (at days 7 and 9 after cancer cell implantation) significantly suppressed tumor growth in the xenograft mice (Figure 5f,g). In both the ovarian and breast cancer xenograft models, AD‐delivered NC saRNA did not have any significant effects on tumorigenesis or tumor growth.

Finally, we verified the effects of saMAS1+1982/AD in pancreatic cancer organoids derived from patient tumor specimens from the PaCaOmics cohort (ClinicalTrials.gov registration no. NCT01692873).^[^ ^16^ ^]^ Consistent with the ovarian and breast cancer models, treatment with saMAS1+1982/AD significantly enhanced *MAS1* gene expression (Figure S8, Supporting Information) and reduced the viability of cells in the organoids (Figure 5h). Of note, there was no significant change when the organoids were treated with the NC saRNA/AD complex. Taken together, our data suggest that the identified saMAS1/AD complex has potential for the treatment of various cancer types.

To further assess the application potential of the identified saRNA targeting MAS1, we also evaluated the in vivo toxicity via histological analysis on the major organs, and indexes of liver and kidney functions on mice treated with saRNA/AD after either i.p. or i.v. injections. Notably, there is no significant changes of the serum transaminase (aspartate transaminase [AST), alanine transaminase (ALT), creatinine (CRE), and blood urea nitrogen (BUN), highlighting that the liver and kidney function well after treatment with saRNA/AD (Figures S9a–d and S10a–d, Supporting Information). In addition, there are no significant morphological changes in histological analysis (Figures S9e and S10e, Supporting Information). All these results indicate that the saRNA/AD is safe and biocompatible, devoid of any notable adverse effect for future application in treating cancer.

## 3 Discussion

The concept of saRNA‐mediated gene activation was first described in 2006.^[^ ^5^ ^]^ saRNA technology provided a new and promising tool for selectively promoting gene transcription in precision medicine.^[^ ^17^ ^]^ However, saRNA has garnered attention only recently after the successful clinical trial of the first saRNA drug candidate (MTL‐CEBPA) for advanced liver cancer.^[^ ^7^, ^18^ ^]^ Herein, we show that saRNAs developed against *MAS1* and delivered by the dendrimer vector AD can consistently upregulate *MAS1* expression in multiple cancer cell lines, xenograft models, and patient‐derived organoids. Functionally, saMAS1 suppresses the migration of cancer cells, inhibits tumorigenesis in xenograft models, and impedes the growth of patient tumor‐derived organoids.

**Figure 6:**
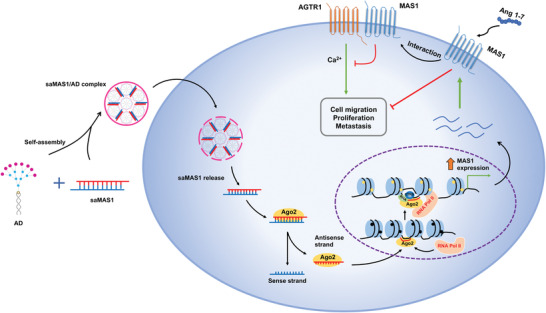
Schematic diagram summarizing the molecular mechanism by which saMAS1/AD enhances *MAS1* gene expression and suppresses cancer progression and metastasis.

MAS1 is a GPCR and acts as an inhibitory regulator of cancer.^[^ ^19^ ^]^ It has been suggested that activation of the Ang 1–7/MAS1 axis could represent a promising therapeutic strategy.^[^ ^20^ ^]^ Other than activation by the endogenous MAS1 ligand, Ang 1–7, recent studies indicate the existence of an Ang 1–7‐independent mechanism by which MAS1 counteracts AGTR1 functions.^[^ ^14^, ^21^ ^]^ Here, we demonstrate the signaling properties and conformation changes of MAS1/AGTR1 heterocomplexes. Our results indicate that the saRNA‐enhanced MAS1 expression counteracts the Ang II/AGTR1 axis via both ligand‐dependent and ‐independent mechanisms, and is beneficial to suppress cancer development in animal models (**Figure** **6**). Our study therefore highlights the tremendous potential of using and leveraging saRNAs to address complex target such as MAS1 as novel therapy for the management of cancers, which currently have limited treatment options. We also anticipate that it will be feasible to modulate other physiological and pathological pathways using a similar approach. Because of the pleiotropic role of the RAS in multiple physiological processes, the MAS1‐targeting saRNAs developed in this study may also be extended to treat other diseases, such as cardiovascular^[^ ^13a^ ^]^ or COVID‐19‐associated diseases.

Compared to siRNA, which is the predominant method for silencing gene expression in the field of molecular biology, there are limited approaches for upregulating gene expression. Classic methods, such as direct delivery of DNA and mRNA, have several disadvantages. These include difficulties in nucleic acid synthesis and delivery as well as potential immunogenicity. The development of saRNA technology has provided a new tool for selective RNA regulation and is easier to implement in practice. Further, when compared to conventional chemical agonist/antagonist agents, saRNAs are particularly promising for providing a straightforward and highly specific approach to target GPCRs, because GPCRs are often considered a difficult class of drug targets due to their complex structure, various subtypes, and activation mechanism. This first report of a saRNA approach to regulating GPCRs represents a novel strategy and opens a new avenue to effectively targeting GPCRs, thus expanding the druggable “GPCRome.” We are working actively in this direction.

## 4 Experimental Section

### Design of saRNA

The design of saRNA was based on a bioinformatics algorithm as described previously^[^ ^22^ ^]^ or in accordance with standard protocols of saRNA design.^[^ ^23^ ^]^ The latter includes location‐related rules and duplex‐related rules. In brief, repeat elements and CpG islands were avoided. The size of the saRNA duplex was ≈21 nucleotides with GC content in the saRNA ranging between 40% and 60%. The [dT][dT] overhangs were added to the 3′‐terminal of the saRNA duplex. To bind the argonaute (Ago) protein, the duplex required low thermodynamic stability at the 5′‐end. The saRNA sequence targeting MAS1 is listed in Table S1, Supporting Information. All saRNAs were synthesized by Sangon Biotech (Shanghai, China). The saMAS1+1982 expressing biotin conjugated at the 3′ end (biotinylated‐saRNAs) was synthesized for the ChIbRP assay.

### Cells Culture

Four ovarian cancer cell lines (A2780, OVCA429, JHOM2B, and SKOV‐3), 2 breast cancer cell lines (MDA‐MB‐231 and HCC1937), and three pancreatic cancer cells (AsPC‐1, PANC‐1, and CFPAC1) were used. For receptor interaction and cell signaling studies, HEK293 cells were used. The ovarian cancer, breast cancer, and HEK293 cell lines were cultured in Dulbecco's modified Eagle's medium (DMEM) supplemented with 10% fetal bovine serum (FBS), 100 U mL^−1^ of penicillin and 100 µg mL^−1^ of streptomycin (Gibco Laboratories, Gaithersburg, MD, USA). AsPC‐1 cells were cultured in Roswell Park Memorial Institute 1640 medium supplemented with 10 mM of HEPES; PANC‐1 cells were cultured in DMEM, and CFPAC‐1 was cultured in Iscove's modified Dulbecco's medium. All pancreatic cell lines were supplemented with 10% FBS, 100 U mL^−1^ of penicillin and 100 µg mL^−1^ of streptomycin. All cell lines were cultured in a cell incubator with 5% CO_2_ at 37 °C.

### Organoids

Primary pancreatic cancer organoids were obtained from consecutive patients under the Paoli Calmettes Institute clinical trial NCT01692873 (https://clinicaltrials.gov/show/NCT01692873). Single consent's forms were collected from informed patients and registered in a central database. A total of 3.0 × 10^3^ single organoid cells per well were re‐seeded in ultra‐low‐attachment 96‐well plates (Costar; Corning Inc., Corning, NY, USA). After overnight culture, they were then treated with saRNA/dendrimer complexes (50 nm of saRNA, N/P = 10, with N and P denoting the number of the terminal amine groups in AD and the number of phosphate groups in saRNA, respectively) for 7 days. Each experiment was performed in triplicate and repeated more than or equal to two times.

### TEM Imaging

TEM studies were performed using a JEOL JEM‐TEM system (JEOL, Tokyo, Japan) to characterize the size and morphology of the saRNA/AD complex at an accelerating voltage of 200 kV. As described previously, a solution of saRNA was mixed with a solution of dendrimer AD in Milli‐Q water at the required concentration and at the N/P ratio of 10. After equilibration (30 min), 4.0 µL of this mixture was dropped on a standard carbon‐coated copper TEM grid, then allowed to evaporate (30 min at 30 °C, ambient pressure). The grid was then stained with 3.0 µL of uranyl acetate (2.0% in aqueous solution) for 5 s, and the excess of uranyl acetate was removed using filter paper before measurements. Data were analyzed with the Digital Micrograph software.

### DLS Analysis

The saRNA solution was mixed with the AD solution in H_2_O at N/P ratio of 10. Following incubation at 25 °C for 24 h, size distribution and zeta potential measurements were performed using the Zetasizer Nano‐ZS system (Malvern, Ltd. Malvern, UK) with a He–Ne ion laser of 633 nm. The experiments were performed in triplicate.

### Gel Retardation Analysis

To confirm the ability of the AD to form stable complexes with saRNA, a gel retardation assay was developed and naked saRNA served as a control. The saRNA/AD complexes were prepared at different N/P ratios ranging from 0.4 to 15 in phosphate‐buffered saline (PBS). After being incubated at 37 °C for 30 min, complexes with equal amounts of saRNA were then loaded on 1.2% agarose gels. Electrophoresis was performed in Tris/borate/ethylenediaminetetraacetic acid (EDTA) (TBE) buffer at 100 V for 20 min with saRNA bands visualized using ethidium bromide, then detected using an EASY CCD camera (type 429K) (Herolab, Wiesloch, Germany).

### saRNA/Dendrimer Complexes Stability to RNase A and Serum

To assess the ability of the dendrimer AD to protect saRNA from RNase degradation and in serum, the saRNA stability assay was performed. The saRNA/AD complexes were prepared at an N/P ratio of 10 based on the gel retardation assay. The complexes were then incubated with either 0.25 µg mL^−1^ of RNase A solution or 5–10% FBS solution at 37 °C, with free saRNA serving as a control. An aliquot of sample was collected at different time intervals (0, 5, 10, 20, 30, 60, 90, and 120 min). The aliquoted samples were mixed with 0.5% sodium dodecyl sulfate (SDS) to inactivate RNase A and kept on ice. Samples were then loaded on 2.0% agarose gels with TBE buffer at 100 V for 20 min with saRNA bands visualized using ethidium bromide. The visible bands were detected by a Herolab EASY CCD camera (type 429K) (Herolab, Wiesloch, Germany), which indicated saRNA stability.

### Dendrimer‐Mediated saMAS1 Delivery

The AD was synthesized as described previously.^[^ ^24^ ^]^ To form the saRNA/AD complex, a solution of saRNA was simply mixed with a solution of dendrimer AD at the required concentrations and the desired N/P ratios. Specifically, the stock solutions of saRNAs and dendrimer AD were diluted in Opti‐MEM (Thermo Fisher Scientific, Waltham, MA, USA) separately at the required concentrations and incubated for 10 min at room temperature. saRNAs were then mixed with dendrimer AD and incubated for another 30 min at room temperature.

In brief, cells were seeded at 2.0 × 10^5^ cells per well in a 6‐well plate. Also, 3.0 × 10^5^ organoid single cells were seeded per well in Matrigel‐coated 6‐well plates. After overnight culture, the prepared complexes were added to the cells or organoids dropwise, and the cells or organoids were incubated at 37 °C in a 5% CO_2_ incubator for 3–7 days, respectively.

### Lipofectamine‐Mediated saMAS1 Delivery

For Lipofectamine‐mediated saRNA delivery, cells were seeded in a 6‐well plate 24 h before transfection. Lipofectamine 3000 was mixed with saMAS1 (50 nM) in a ratio of 7.5 (Lipofectamine:RNA = 7.5:1, v/w) (Invitrogen, catalog no. L3000150) and were applied to the target cells according to the manufacturer's protocol. 6 h after the treatment, the cells were re‐supplied with the complete culture medium.

### RNA Isolation and Quantitative Real‐Time PCR

The effects of saRNA were evaluated by quantitative real‐time PCR (RT‐qPCR). After transfection, total RNA was extracted by TRIzol reagent (Invitrogen, Carlsbad, CA, USA). Then, total RNA (2 µg) was reverse‐transcribed by a SuperScript IV First‐strand synthesis system (Invitrogen). The relative level of transcripts was determined by Fast SYBR Green Master Mix (Applied Biosystems, Foster City, CA, USA). PCR amplification and detection of fluorescence signals were performed in an ABI 7500 Fast Real‐time PCR system (Applied Biosystems). The sequences of PCR primers are listed in Table S2, Supporting Information. Glyceraldehyde 3‐phosphate dehydrogenase was used as the house‐keeping gene to determine the relative expression of target genes using the 2^−ΔΔCt^ method.^[^ ^25^ ^]^

### Western Blotting

Cells were harvested 48 h after transfection for protein extraction with 0.20 mL of lysis buffer (50 mm Tris, pH 7.4, 0.25% Na‐deoxycholate, 1.0% NP‐40, 150 mm NaCl, 1.0 mm EDTA, and 1.0% *β*‐mercaptoethanol, with cOmplete proteinase inhibitor [Roche Holdings, Basel, Switzerland]). Extracts were sonicated (Bioruptor; Diagenode, Liege, Belgium) at a high‐power for ten cycles (30‐s on/off for each cycle). The protein concentration was determined using Coomassie Brilliant Blue (Bio‐Rad Laboratories, Hercules, CA, USA). SDS‐polyacrylamide gel electrophoresis using 10–12% gels was employed to separate 20–30 µg of total protein. Proteins were transferred to polyvinyl‐difluoride membranes (Bio‐Rad Laboratories). The antibodies used in western blotting assays are listed in Table S3, Supporting Information. Clarity Western ECL substrate (Bio‐Rad Laboratories) and the ChemiDoc XRS+ imaging systems (Bio‐Rad Laboratories) were used for signal detection.

### Confocal Imaging

SKOV‐3 cells were seeded in a 35‐mm imaging dish with a polymer coverslip bottom (Ibidi, Gräfelfing, Germany) for 24 h. FAM‐labeled RNA (FAM‐saMAS1+1982) were delivered into the cells by AD. After a 4 h of incubation, the cells were washed with PBS twice. The nuclear DNA was stained with 1:1000 Hoechst 33342 solution. The fluorescence signal of FAM‐saMAS was captured with an LSM710 confocal microscope (Carl Zeiss, Jena, Germany) where the FAM: excitation/emission was 488/506 nm and Hoechst 33342: excitation/emission was 352/455 nm. For PANC‐1 cells, the cells were seeded in a 12‐well plate at a density of 1.0 × 10^5^ cells/well for 24 h, then incubated with the Cy3‐saRNA (Cy3‐saMAS1+1982, 50 nm)/AD complexes at N/P ratio 10 for 4 h. The cells were washed three times with PBS, and then Hoechst33342 (1.0 µg mL^−1^) was added, and stained for 30 min at 37 °C. The cells were visualized using a Zeiss LSM880 Meta laser scanning confocal microscope (Carl Zeiss, Jena, Germany). Naked saRNA were used as the controls.

### Flow Cytometry Assay for AD‐Mediated Delivery of saRNA

A2780 cells were first seeded in a 6‐well plate for 24 h. Then, Cy5‐labeled saMAS1+1982 with AD complexes was used to treat the cells for 4 h. The cells were washed with 1× PBS for three times and harvested by Tryspin/EDTA. Fluorescence‐activated cell sorter (FACS) analysis was performed to detect the fluorescence signal by CytoFlex S (Beckman, Coulter, Brea, CA, USA). At least 10 000 events were counted in each data point and repeated in triplicate. For PANC‐1 with Cy3‐labeled RNA, FACS were performed using MACS flow cytometry (Miltenyi Biotec, Surrey). PBS and naked saRNA were served as the controls. All experiments were performed in triplicate. The data were analyzed using FlowJo software (FlowJo, LLC).

### ChIbRP Assay

The ChIbRP assay was conducted according to the method described by Voutilla et al.^[^ ^26^ ^]^ In brief, A2780 cells were transfected with biotinylated saRNA by dendrimers. After 24 h, the transfected cells were cross‐linked with 1.0% formaldehyde for 10 min at room temperature. The reaction was quenched by 250 mm glycine for 3 min. After washing three times with ice‐cold PBS, cells were lysed by RIPA buffer (150 mm NaCl, 1.0% NP40, 0.50% sodium deoxycholate, 0.10% SDS, and 50 mm Tris). Extracts were sonicated by a sonicator (Diagenode) at high power for 30 cycles (30‐s on/off for each cycle). The ideal size of a chromatin fragment was about 200–300 bp as confirmed by agarose gel electrophoresis. Biotin immobilization was undertaken overnight on a rotating chamber at 4 °C using a magnetic Dynabead biotin binder (Invitrogen). Afterward, the beads were washed by low‐salt buffer (0.10% SDS, 1.0% Triton X‐100, 2.0 mm EDTA, 20 mm Tris‐HCl, and 150 mm NaCl_2_), high‐salt buffer (0.10% SDS, 1.0% Triton X‐100, 2.0 mm EDTA, 20 mm Tris‐HCl, and 500 mm NaCl), lithium chloride buffer (0.25 m LiCl, 1.0% NP40, 1.0% deoxycholate, 1.0 mm EDTA, and 10 mm Tris‐HCl), and Tris‐EDTA (TE) buffer. The cross‐links were reversed for 4 h at 65 °C with 300 mm NaCl. The precipitated DNA was purified by phenol/chloroform/isoamyl alcohol extraction and ethanol precipitation. Finally, the pellets were resuspended in elution buffer (10 mm Tris‐Cl, pH 8.5). The target *MAS1* fragment was amplified by SYBR Green PCR Master Mix (Applied Biosystems). The primers used are listed in Table S2, Supporting Information.

### BRET Assay

For the saturation BRET assay, HEK293 cells were co‐transfected with a fixed amount of nanoluciferase‐tagged AGTR1 (0.25 µg per well in a 6‐well plate) and increasing amounts of MAS1‐YFP (0–2.0 µg per well) added. BRET measurements were determined as described previously.^[^ ^27^ ^]^ Briefly, 48 h after transfection, cells were detached by Versene Solution (Thermo Fisher Scientific) and re‐seeded in black 96‐well plates at 100 000 cells per well. The luciferase substrate, coelenterazine h (final concentration, 5.0 µm), was added, and the luminescence signal was measured using an Envision multiplate reader (PerkinElmer, Waltham, MA, USA). The BRET value was calculated based on the ratio of long‐wavelength (510 nm)/short‐wavelength (485 nm) emission signals.

### Cell‐Signaling Assay

For the SRE assay, HEK293 cells were seeded in 48‐well plates at 1.4 × 10^4^ cells per well. Cells were transfected transiently with 75 ng of reporter vectors (pGL4.33[*luc2P*/SRE/Hygro]) and 75 ng of AGTR1 and/or MAS1 expression vector(s) (pcDNA 3.1. was used to balance the total amount of DNA). Next, 24 h after transfection, cells were incubated with Ang II for 6 h at 37 °C before assaying for luciferase activity. The latter (relative luminescence units) was determined using the One‐Glo Assay kit (Promega Corporation, Madison, WI, USA) and an Envision multiplate reader (PerkinElmer, Waltham, MA, USA). For the calcium assay, HEK293 or OVCA429 cells were transiently transfected transiently with 2.0 µg of AGTR1 with or without MAS1 expression vector(s) (the total DNA was balanced by pcDNA3.1). After 24 h, cells were re‐seeded into 96‐well plates and incubated overnight. For the saMAS1/AD treatment, the cells were treated with saMAS1/AD+1982 (50 nm) for 24 h before the assay. The calcium assay was conducted following the protocol of the FLIPR Calcium 5 assay kit (Molecular Devices, Silicon Valley, CA, USA). Ang II (100 nm) was used to stimulate cells.

### Intramolecular FlAsH Assay

FlAsH probes of AGTR1 with a tetracysteine tag (CCPGCC) incorporated into ICLs were kindly provided by Prof. Sun Jinpeng (Shandong University, Shandong, China).^[^ ^28^ ^]^ HEK293 cells were transfected with the tetracysteine tag AGTR1 with or without a MAS1 expression vector. After transfection into HEK293 cells, CCPGCC sequences on the AGTR1 probes were labeled using the TC‐FlAsH II in‐cell tetracysteine tag detection kit (Thermo Fisher Scientific) according to the manufacturer's protocol. Labeled cells were treated with Ang II for 15 min at room temperature before the BRET assay. The intramolecular BRET assay was measured using the Envision multiplate reader.

### Wound‐Healing Assay

After 24 h of transfection, cells were scratched gently with a P200 pipette across the center of each well along the ruler. Then, cells were washed three times with warm HBSS to remove scattered cells. Next, 0.10 mL of HBSS was added and images of wound healing were taken 24, 48, and 72 h later using a light microscope (EVOS; Thermo Fisher Scientific). The migration distance was measured using ImageJ (U.S. National Institutes of Health, Bethesda, MD, USA).

### Apoptosis/Necrosis Assay

The OVCA429 cells were treated with saMAS1/AD for 24 h before being re‐seeded on a 100‐mm dish to allow pre‐coated with agarose the spheroid formation. After 72 h, the spheroids were collected and resuspended in 1× PBS to obtain suspended cells. The cells were stained with Annexin V FITC and propidium iodide (Annexin V apoptosis detection kit FITC; Sangon Biotech). The stained cells were analyzed by flow cytometry (BD Accuri C6 flow cytometer, with ≥10 000 events; Becton Dickinson, Franklin Lakes, NJ, USA).

### Organoid Viability Assay

The CellTiter‐Glo 3D cell viability assay (Promega Corporation) was utilized to evaluate organoid's proliferative ability according to the manufacturer's instruction. Briefly, an equivalent volume of CellTiter‐Glo 3D reagent was added to all samples and shaken at room temperature for 60 min. The content in the wells was then moved into an assay plate, and their bioluminescence activity was appraised by a plate reader. Later, organoids viability was computed according to the standard curve.

### RT‐qPCR of Organoids

Total RNA was obtained from harvested organoids after 48 h of transfection with saRNA/dendrimer complexes by using a RNeasy kit (Qiagen, Hilden, Germany), and the GoScript reverse transcriptase kit (Promega Corporation) was used for reverse transcription. qPCR was performed on a MXPro‐MX3005P qPCR system (Stratagene, La Jolla, CA, USA). House‐keeping gene 36B4 was used for normalization and quantification. The sequences of the primers used in this study are presented in Table S2, Supporting Information.

### Ovarian and Breast Cancer Xenograft Models

Female NOD/SCID mice (aged 4–6 weeks; Charles River Laboratories, Wilmington, MA, USA) were used to create the xenograft model. The animal experiments were approved by the Animal Research Ethics Committee of the University of Macau (UMARE‐019‐2019) in Macau, China. AD was effective for in vitro delivery at N/P ratios of both 5 and 10. The results at an N/P ratio of 10 were often slightly better than those at an N/P ratio of 5 for cell‐based in vitro assay. An N/P ratio of 5 was used to reduce the amount of the vector and the potential adverse effect in the xenograft models. For the ovarian cancer model, A2780 cells (2 × 10^6^) were suspended in 0.20 mL of HBSS and then injected into the peritoneal cavity. 1 day after injection, mice were separated randomly into different groups for different drug treatments. For each group, ten mice were sacrificed. For treatment with saMAS1/AD, a dendrimer mixture (1.0 mg kg^−1^ saRNA, N/P ratio = 5) was injected by intraperitoneal injection (i.p.) 1, 3, and 5 days after the injection of cancer cells. Mice were sacrificed on day 26. The number and volume of tumors were calculated.

For breast cancer xenografts, the experiments were approved by the Animal Research Ethics Committee of the University of Macau (UMARE‐045‐2020) in Macau, China. Briefly, 4–6‐week‐old nude mice were used. MDA‐MB‐231 cells (6 × 10^5^) were resuspended with 100 µL 50% Matrigel, and then subcutaneously injected in the right flanks of the mice. After 7 days, the tumors were observed. Small RNA‐dendrimer treatment (1.0 mg kg^−1^ saRNA, N/P ratio = 5) was carried out every other day by in situ subcutaneously injection, for a total of two injections. For monitoring tumor growth, the tumor volume was measured every 4 days.

### In Vivo Toxicity Evaluation

For in vivo toxicity assay, PBS, NC/AD, or saRNA/AD (1.0 mg kg^−1^ saRNA, N/P ratio = 5) were injected into balb/c mice (aged 6–8 weeks) via the intraperitoneal injection and tail vein injection (*N* = 3 for each injection). The animal experiments were approved by the Animal Research Ethics Committee of the University of Macau (UMARE‐AMEND‐137) in Macau, China. After 24 h injection, serum samples were collected. The concentrations of AST, ALT, CRE, and BUN were measured by corresponding detection kits, and the operation steps strictly followed the protocol provided by the supplier (Nanjing Jiancheng Bioengineering Institute, China).

### Histological Analysis

The histological changes of major organs including heart, liver, spleen, lung, and kidney were analyzed for mice after treatment with PBS, NC/AD, or saRNA/AD (1.0 mg kg^−1^ saRNA, N/P ratio = 5) for 24 h. The major organs were fixed by paraformaldehyde (4%) and embedded in paraffin. Then, they were sliced into 5 µm sections for H&E staining.

### Statistical Analyses

Data were represented as the mean ± SEM values of ≥3 independent experiments. Differences between groups, the sigmoidal curves of the reporter luciferase assay, and the saturation curves of the BRET assay were analyzed using by one‐phase association exponential equation in GraphPad Prism version 5.01 (GraphPad Software, San Diego, CA, USA). Data with two groups were analyzed by Student's *t*‐tests. Data with ≥3 groups were analyzed by one‐way analysis of variance (ANOVA). *p* < 0.05 was considered to indicate a significant difference and the detail of such are indicated in the figure legends.

## Conflict of Interest

N.H. declares financial interests in MiNA Therapeutics. Other authors declare no conflict of interest.

## Author Contributions

L.T.O.L. and L.P. conceived the idea, designed the study, analyzed the data, and directed the project. N.H. designed saRNAs. Y.X. performed the experiments and analyzed data. W.L., T.R., Y.J., X.Z., and J.W. helped in preparation and characterization of the saRNA/AD complexes: T.R. performed TEM and DLS experiments, W.Y. and J.W. evaluated cellular uptake, and W.L. assessed the stability and realized all studies related to patient pancreatic cancer models. N.D. and J.I. provided support and materials for the pancreatic organoid experimental assay. Q.Z. and R.K. provided support in cell signaling assays. A.S.T.W., J.S.S., and R.X. provided materials and help in xenograft experiments. Y.X., L.P., and L.T.O.L. wrote the manuscript with the approval of all the co‐authors.

## Supporting information

Supporting Information

## Data Availability

The data that support the findings of this study are available from the corresponding author upon reasonable request.
