# Supplementary material for: Small Activating RNA Modulation of the G Protein‐Coupled Receptor for Cancer Treatment
Source: Adv Sci (Weinh). 2022 Jun 16;9(26):2200562. doi: 10.1002/advs.202200562 (PMC9475523; doi:10.1002/advs.202200562)
Supplement: Supplementary file 1 — Supporting Information [file ADVS-9-2200562-s001.pdf]

ADVANCED SCIENCE
Open Access

Supporting Information

for *Adv. Sci.*, DOI 10.1002/advs.202200562

Small Activating RNA Modulation of the G Protein-Coupled Receptor for Cancer Treatment

Yunfang Xiong, Ran Ke, Qingyu Zhang, Wenjun Lan, Wanjun Yuan, Karol Nga Ieng Chan, Tom Roussel, Yifan Jiang, Jing Wu, Shuai Liu, Alice Sze Tsai Wong, Joong Sup Shim, Xuanjun Zhang, Ruiyu Xie, Nelson Dusetti, Juan Iovanna, Nagy Habib, Ling Peng and Leo Tsz On Lee**

Supplementary Figure 1

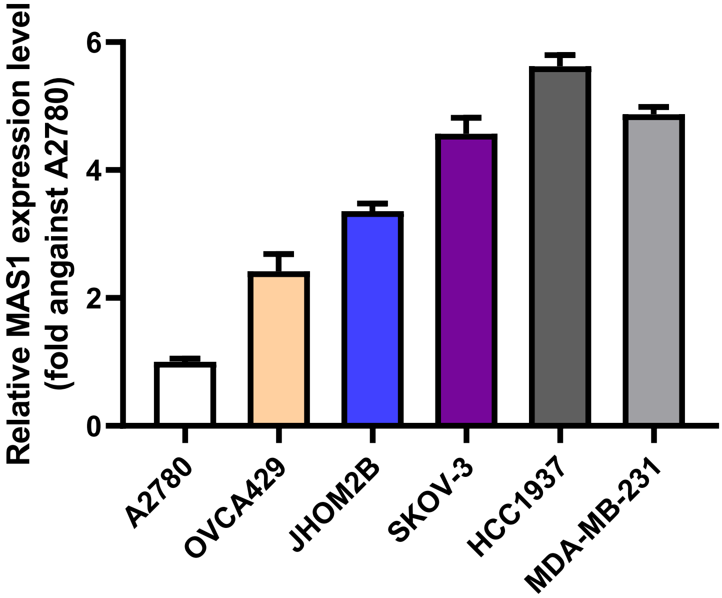

Supplementary Figure 1. Endogenous expression of the *MAS1* gene in ovarian and breast cancer cell lines. The expression levels of *MAS1* were detected by real-time qPCR. The relative expression was calculated by the $2^{-\Delta\Delta Ct}$ method, with the level in A2780 defined as 1.

Supplementary Figure 2

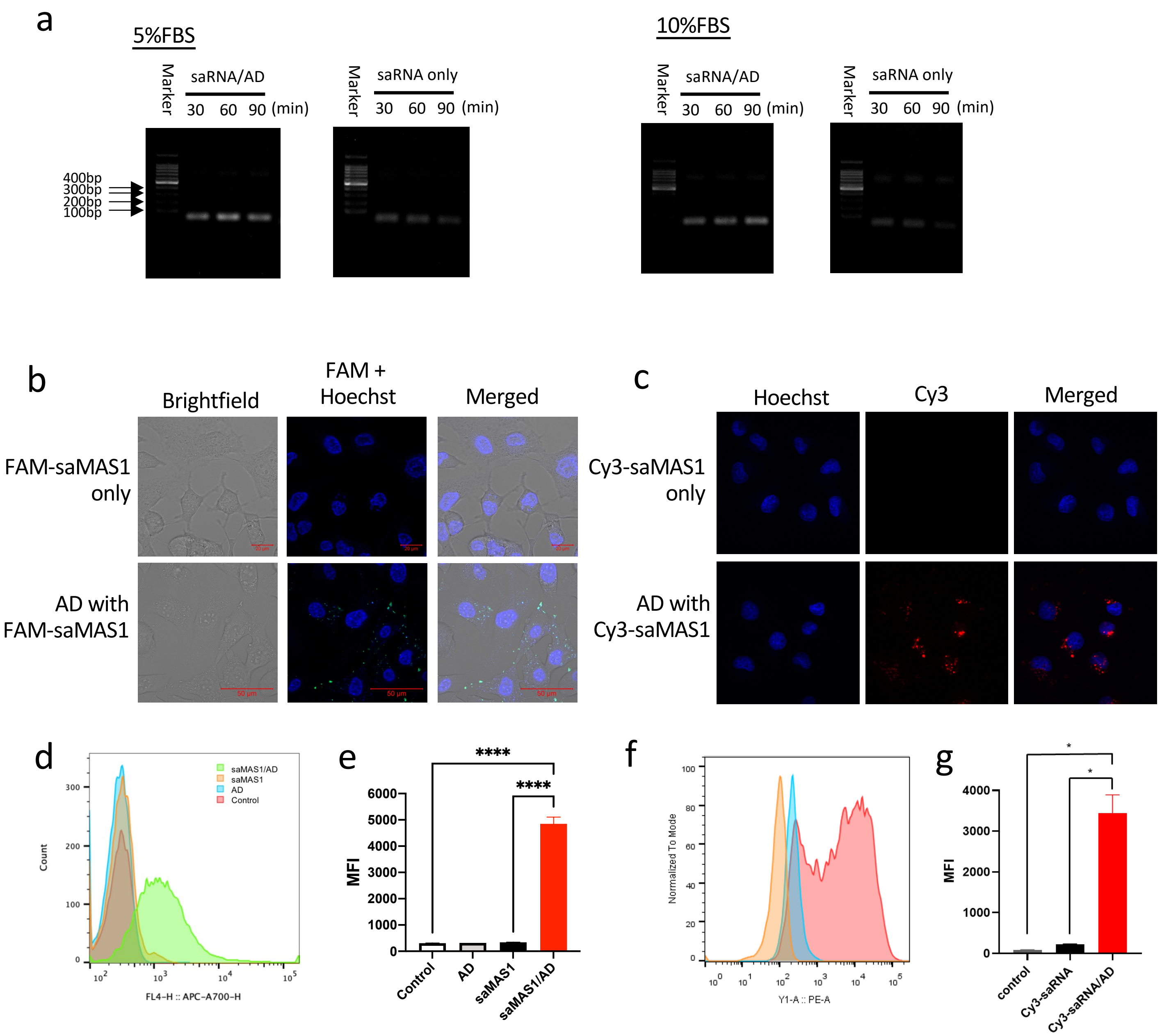

Supplementary Figure 2. (a) The saRNA/AD complexes protect saMAS1+1982 (saRNA) from degradation by serum in solution with 5% FBS (left) or 10% FBS (right). (b-c) Cellular uptake of saRNA with and without the dendrimer vector AD was studied by confocal microscopy using saRNA labelled with fluorescence dye FAM or Cy3. (b) Confocal fluorescent images on the uptake of the FAM-labelled saRNA into SKOV-3 cells in the presence and absence of AD. Blue: Hoechst 33342; green: FAM-labelled RNA. (c) Confocal fluorescent images on the uptake of Cy3-labelled saRNA into PANC-1 cells. Blue: Hoechst 33342; red: Cy3-labelled RNA. (d-g) Flow cytometry analysis of the cellular uptake of saRNA with or without the dendrimer vector AD. (d) Flow cytometry results about the Cy5-labelled saMAS1(+1982)/AD uptake in ovarian cancer A2780 cells. (e) The Cy5 signals are represented as Mean Fluorescence Intensity (MFI). (f) Cy3-labelled saMAS1(+1982)/AD uptake by PANC-1 cells. (g) The Cy3 signals in PANC-1 cells are represented as MFI. All the data in this figure are presented as mean ± SEM values in triplicate. P-values are calculated by using one-way ANOVA with Dunnett correction. Significant differences are indicated as *$P < 0.05$; **** $P < 0.0001$ vs. control or saMAS1.

Supplementary Figure 3

Ovarian cancer

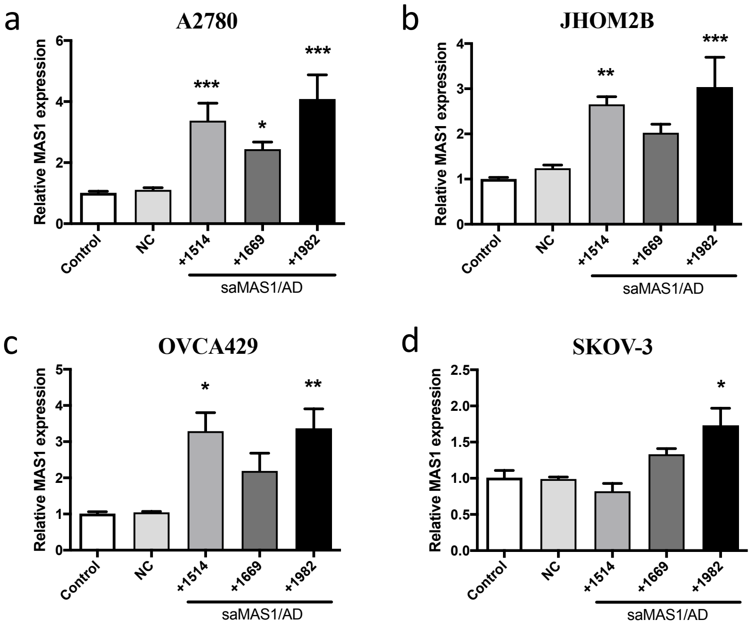

Supplementary Figure 3. Upregulation of *MAS1* mRNA levels by saMAS1/AD in ovarian cancer (a) A2780, (b) JHOM2B, (c) OVCA429, and (d) SKOV-3 cells. Cells were treated with saMAS1/AD carrying 50 nM saRNA at a N/P ratio of 10. *MAS1* expression levels were detected by real-time qPCR. The relative expression was calculated by the $2^{-\Delta\Delta Ct}$ method, with the level in untreated control cells defined as 1. All the data in this figure are presented as mean ± SEM values from at least three experiments. *P* values are calculated by using one-way ANOVA with Dunnett correction. Significant differences are indicated as * $P < 0.05$; ** $P < 0.01$; *** $P < 0.001$ vs. control.

Supplementary Figure 4

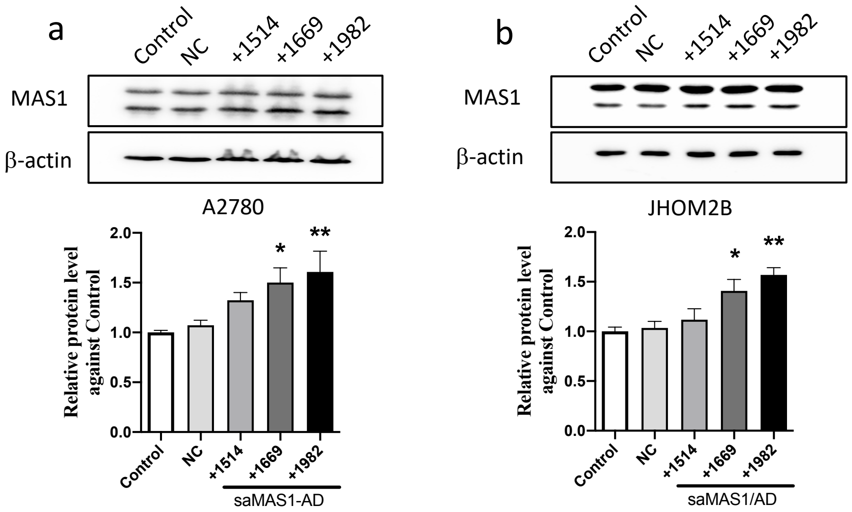

Supplementary Figure 4. saMAS1/AD increases MAS1 protein levels in the ovarian cancer cell lines (a) A2780 and (b) JHOM2B as evaluated by western blot. Upper: representative western blot; lower: quantification of protein levels from 3 experiments. *P* values are calculated by using one-way ANOVA with Dunnett correction. Significant differences are indicated as * *P* <0 .05; ** *P* < 0.01 vs. control.

Supplementary Figure 5

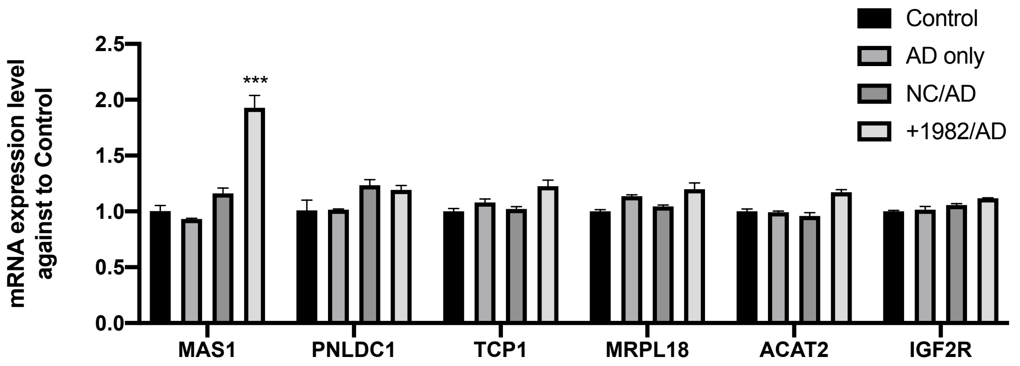

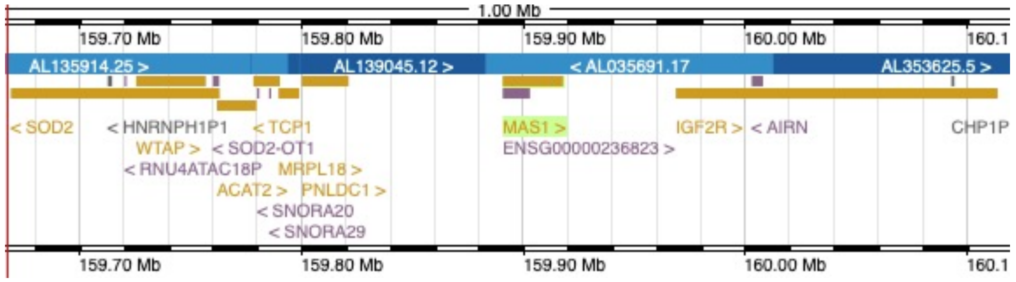

Supplementary Figure 5. The expression of genes near to *MAS1* after treatment of OVCA429 cells with saMAS1+1982/AD. Upper: treatment with saMAS1+1982/AD significantly increases the mRNA levels of *MAS1* but does not affect *PNLDC1*, *TCP1*, *MRPL18*, *ACAT2* and *IGF2R*. Lower: the arrangement of genes on human chromosome 6 near the *MAS1* gene according to the Ensembl database. *P* values are calculated by using one-way ANOVA with Dunnett correction. Significant differences are indicated as *** $P < 0.001$ vs. control.

Supplementary Figure 6

Breast cancer

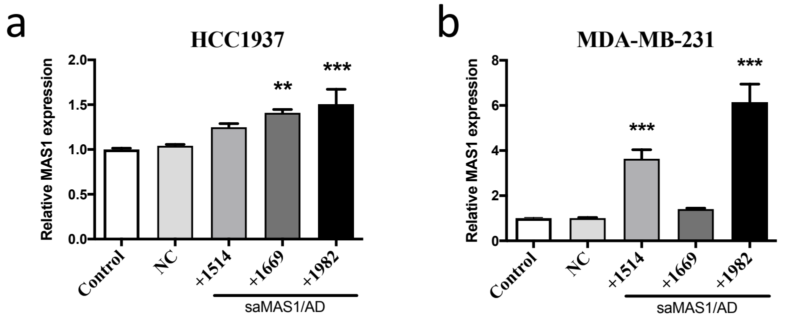

Supplementary Figure 6. Activation of *MAS1* gene expression in breast cancer (a) HCC1937 and (b) MDA-MB-231 cells by saMAS1/AD. All data in this figure are presented as mean ± SEM values from at least three experiments. *P* values are calculated by using one-way ANOVA with Dunnett correction. Significant differences are indicated as ** $P < 0.01$ and *** $P < 0.001$ vs. control.

Supplementary Figure 7

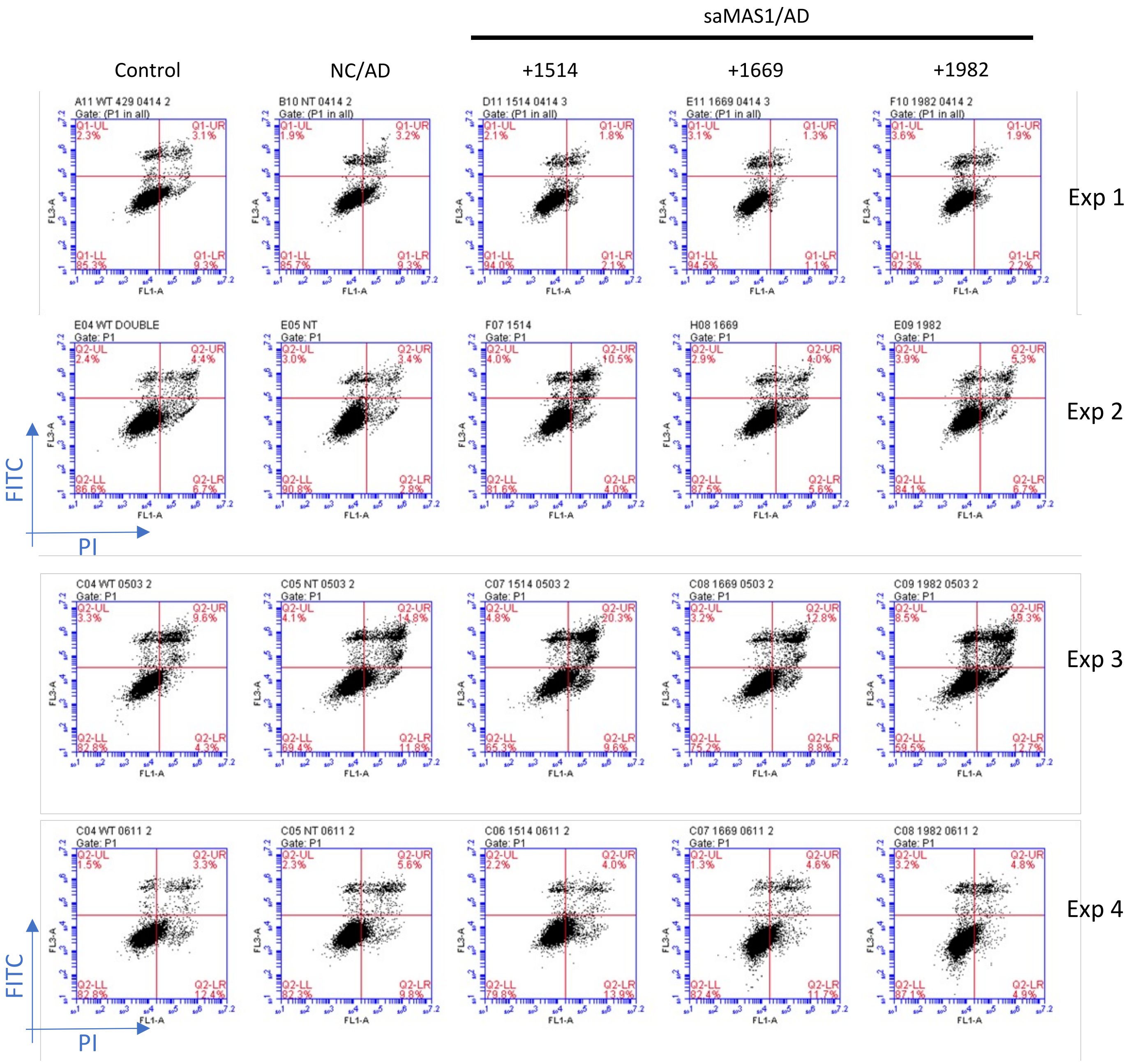

Supplementary Figure 7. saMAS1/AD enhances cancer cell necrosis (Upper Left, UL) and apoptosis (Upper Right, UR + Lower Right, LR). Death of OVCA429 cells in spheroids was assessed by Annexin V-FITC and PI assay using flow cytometry after treatment with non-target saRNA (NC) or saMAS1 delivered by AD. The data from four experiments are shown.

Supplementary Figure 8

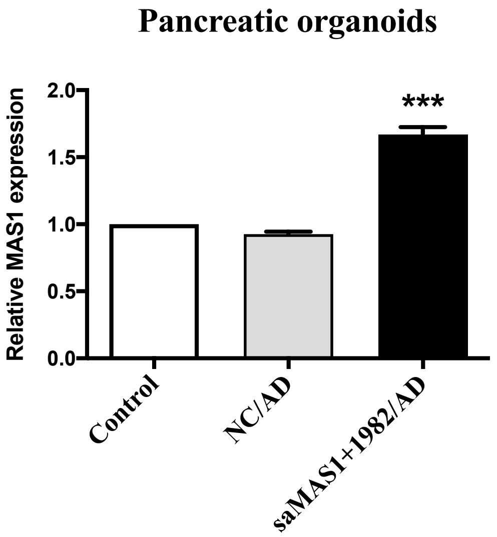

Supplementary Figure 8. saMAS1+1982/AD upregulates the *MAS1* expression level in pancreatic organoids. The mRNA levels of *MAS1* were detected by real-time qPCR. The relative expression was calculated by the $2^{-\Delta\Delta Ct}$ method and the expression level in the un-treatment control was defined as 1. *P* values are calculated by using one-way ANOVA with Dunnett correction. Significant differences are indicated as *** $P < 0.001$ vs control.

Supplementary Figure 9

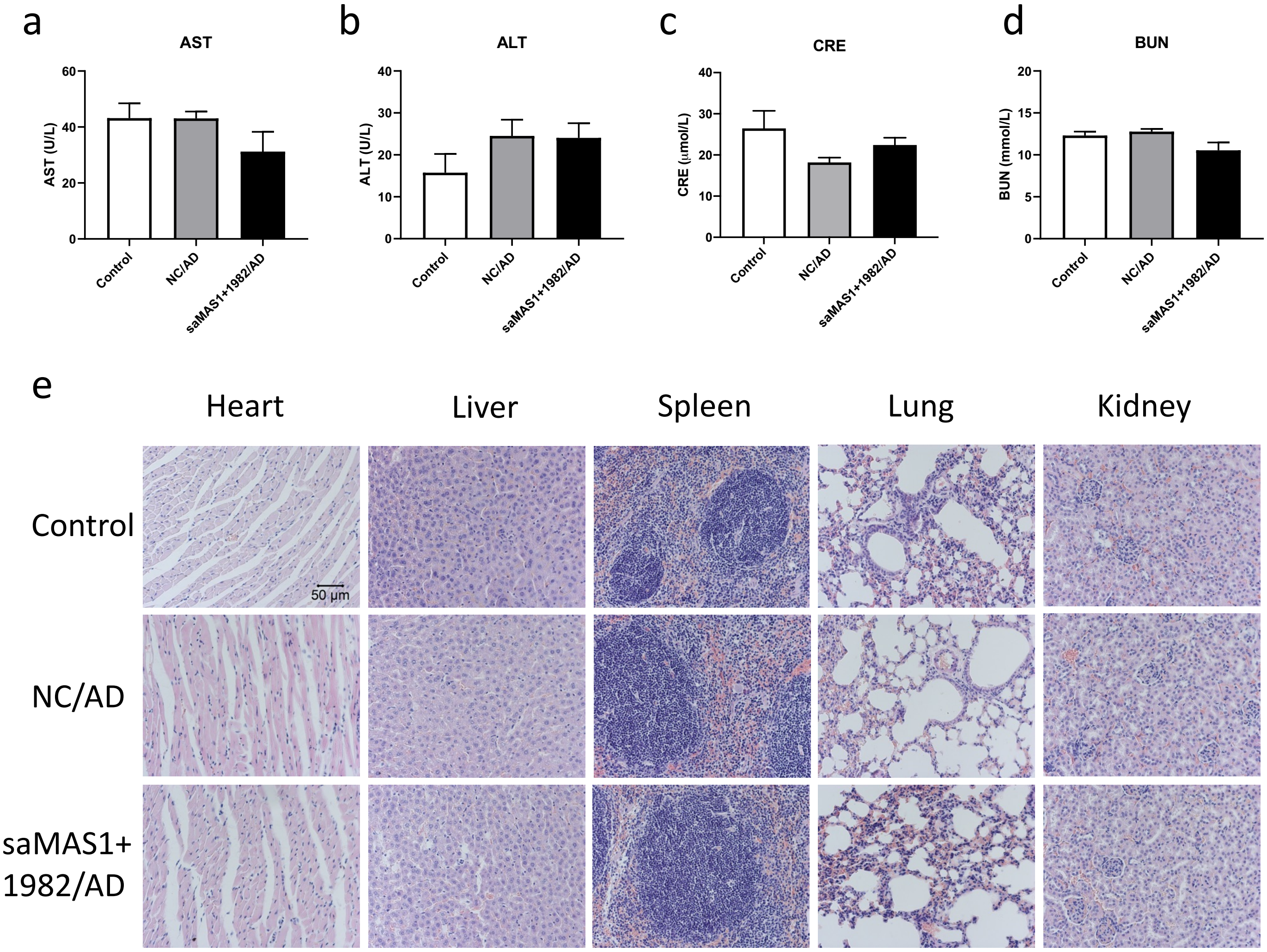

Supplementary Figure 9. *In vivo* toxicity assay of the saMAS1/AD with intraperitoneal injection into Balb/c mice (N=3). (a-d) Major serum biochemistry parameters were measured at 24 h postinjection. Liver function indicators include (a) aspartate transaminase (AST) and (b) alanine transaminase (ALT). Kidney function indicators include (c) creatinine (CRE) and (d) blood urea nitrogen (BUN). P values are calculated by using one-way ANOVA with Dunnett correction. All treatments have no significant change. (e) Histopathological analysis of major organs from mice in control, NC/AD and saMAS1+1982/AD group. Scale bar: 50 μm.

Supplementary Figure 10

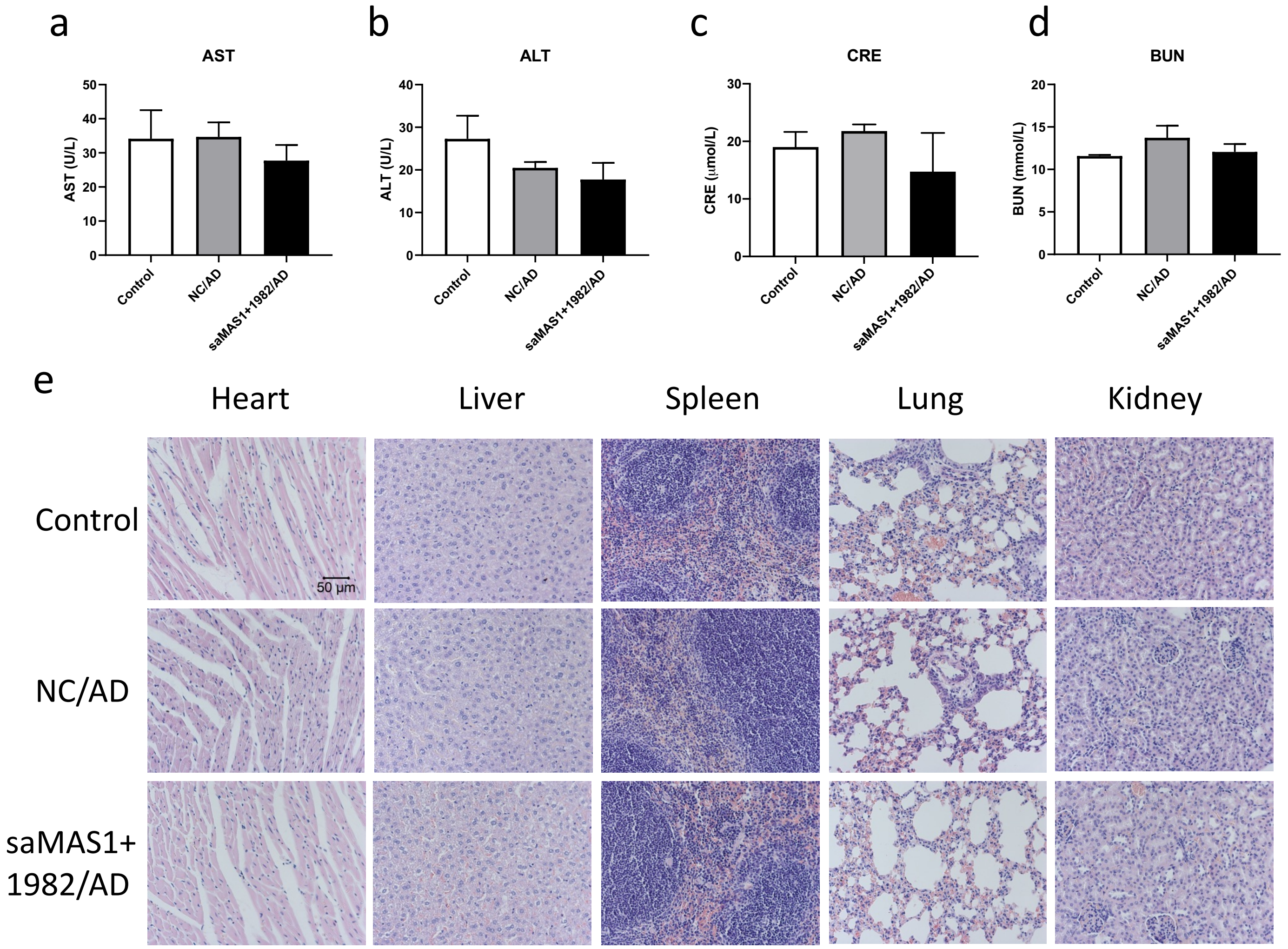

Supplementary Figure 10. *In vivo* toxicity assay of the saMAS1/AD with tail vein injection into Balb/c mice (N=3). (a-d) Major serum biochemistry parameters were measured at 24 h postinjection. Liver function indicators include (a) aspartate transaminase (AST) and (b) alanine transaminase (ALT). Kidney function indicators include (c) creatinine (CRE) and (d) blood urea nitrogen (BUN). P values are calculated by using one-way ANOVA with Dunnett correction. All treatments have no significant change. (e) Histopathological analysis of major organs from mice in control, NC/AD and saMAS1+1982/AD group. Scale bar: 50 μm.

Supplementary Table 1

saRNA oligonucleotides used for MAS1 gene activation.

**saRNA**	**Sequences**
MAS1 -1279 sense<br>MAS1 -1279 antisense	5'-AAUGGUUCUGGUGAUAUUA-dTdT<br>dTdT-UUACCAAGACCACUAUAAU-5'
MAS1 -1259 sense<br>MAS1 -1259 antisense	5'- GUGUUAUUUCGUGGCACUU -dTdT<br>dTdT- CACAAUAAAGCACCGUGAA -5'
MAS1 -573 sense<br>MAS1 -573 antisense	5'- UUGCUUAAAGUGUGAACAA -dTdT<br>dTdT- AACGAAUUUCACACUUGUU -5'
MAS1 -148 sense<br>MAS1 -148 antisense	5'- ACAGUUAUCUUUGUCACAA -dTdT<br>dTdT- UGUCAAUAGAAACAGUGUU -5'
MAS1 +1201 sense<br>MAS1 +1201 antisense	5'- GAAAUAGAGUCUUUGUCAA -dTdT<br>dTdT- CUUUAUCUCAGAAACAGUU -5'
MAS1 +1426 sense<br>MAS1 +1426 antisense	5'- AGACUAUUGCCGCAACCAU -dTdT<br>dTdT- UCUGAUAACGGCGUUGGUA -5'
MAS1 +1514 sense<br>MAS1 +1514 antisense	5'- ACUGAUAUUUGUUGAACAU -dTdT<br>dTdT- UGACUAUAAACAACUUGUA -5'
MAS1 +1669 sense<br>MAS1 +1669 antisense	5'- AGAAGACCUUCGUAAAUUU -dTdT<br>dTdT- UCUUCUGGAAGCAUUUAAA -5'
MAS1 +1708 sense<br>MAS1 +1708 antisense	5'- GGUGUAAUGCCAUGAAAUG -dTdT<br>dTdT- CCACAUUACGGUACUUUAC -5'
MAS1 +1982 sense<br>MAS1 +1982 antisense	5'- UGAGAACUCUGGCUACAAA -dTdT<br>dTdT- ACUCUUGAGACCGAUGUUU -5'
MAS1- +1982-sense<br>MAS1-+1982-antisense-Biotin	5'- UGAGAACUCUGGCUACAAA -dTdT<br>Bio-dTdT- ACUCUUGAGACCGAUGUUU -5'
NC-sense<br>NC-antisense-Biotin	5'-UUCUCCGAACGUGUCACGU-dTdT<br>Bio-dTdT-AAGAGGCUUGCACAGUGCA-5'
FAM-MAS1-+1982-sense<br>MAS1-+1982-antisense	5'- FAM-UGAGAACUCUGGCUACAAA -dTdT<br>dTdT- ACUCUUGAGACCGAUGUUU -5'
FAM-NC-sense<br>NC-antisense	5'-FAM-UUCUCCGAACGUGUCACGU-dTdT<br>dTdT-AAGAGGCUUGCACAGUGCA-5'
Cy5-MAS1-+1982-sense<br>MAS1-+1982-antisense	5'- Cy5-UGAGAACUCUGGCUACAAA -dTdT<br>dTdT- ACUCUUGAGACCGAUGUUU -5'
Cy3-MAS1-+1982-sense<br>MAS1-+1982-antisense	5'- Cy3-UGAGAACUCUGGCUACAAA -dTdT<br>dTdT- ACUCUUGAGACCGAUGUUU -5'

Supplementary Table 2

Primers used in real-time qPCR analysis

**Gene**	**Forward primer**	**Reverse primer**
Real -time qPCR primer		
IGF2R	CCCGAGCTGTGCAGTTATACAT	TGCTGCTCTGGACTCTGTGA
MRPL18	TTGGGGGTTGTTCTCGGTTT	GTTTCATGAGTGGAGGCCGA
TCP1	ATACACAGACATAAGAGGCCAGC	GGGATCCCACCACACAGTTG
ACAT2	ACCTTGACCTTTGCTCAGACC	GTAGGGAATTCCTGCACCCA
PNLDC1	GACTTCGTGGGTCTGGACAT	TGCCTCTCCTTCAATAGCGG
MAS1	TGAGCTTTCTTCTGGCCATT	GACCAATGCCGACTGGTACT
GAPDH	AGGTGAAGGTCGGAGTCA	GGTCATTGATGGCAACAA
ChIbRP primer		
MAS1(+1982)	TGAATTTATGACTTCCTGGGG	TCCTCAGAATCTTTACTCAGC

Supplementary Table 3

The antibodies used for western blot analysis.

Antibody	Vendor	Catalog number	Dilution	Primary/Secondary
MAS1	Santa Cruz Biotechnology	A1419	1:500	Primary antibody
MAS1	Abcam	ab235914	1:1000	Primary antibody
BiP	CST	3177	1:1000	Primary antibody
SREBP	Thermofisher	ma5-11685	1:500	Primary antibody
CHOP	CST	2895	1:1000	Primary antibody
Beta-actin	CST	4970	1:1000	Primary antibody
Goat Anti-Mouse IgG	Bio-Rad	170-6516	1:3000	Secondary antibody
Goat Anti-Rabbit IgG	Bio-Rad	170-6515	1:3000	Secondary antibody
